# Peptide Allergen Immunotherapy: A New Perspective in Olive-Pollen Allergy

**DOI:** 10.3390/pharmaceutics13071007

**Published:** 2021-07-02

**Authors:** David Calzada, Lucía Cremades-Jimeno, María López-Ramos, Blanca Cárdaba

**Affiliations:** 1 Immunology Department, IIS-Fundación Jiménez Díaz-UAM, 28040 Madrid, Spain; davidcalzada87@hotmail.com (D.C.); lucia.cremades@quironsalud.es (L.C.-J.); maria.lramos@quironsalud.es (M.L.-R.); 2 Ciber de Enfermedades Respiratorias (CIBERES), 28029 Madrid, Spain

**Keywords:** T-cell epitopes, synthetic peptides, peptide-allergen immunotherapy, T regulatory response, IL-35, IL-10, vaccines, olive pollen allergy, Ole e 1

## Abstract

Allergic diseases are highly prevalent disorders, mainly in industrialized countries where they constitute a high global health problem. Allergy is defined as an immune response “shifted toward a type 2 inflammation” induced by the interaction between the antigen (allergen) and IgE antibodies bound to mast cells and basophils that induce the release of inflammatory mediators that cause the clinical symptoms. Currently, allergen-specific immunotherapy (AIT) is the only treatment able to change the course of these diseases, modifying the type 2 inflammatory response by an allergenic tolerance, where the implication of T regulatory (Treg) cells is considered essential. The pollen of the olive tree is one of the most prevalent causes of respiratory allergic diseases in Mediterranean countries, inducing mainly nasal and conjunctival symptoms, although, in areas with a high antigenic load, olive-tree pollen may cause asthma exacerbation. Classically, olive-pollen allergy treatment has been based on specific immunotherapy using whole-olive pollen extracts. Despite extracts standardization, the effectiveness of this strategy varies widely, therefore there is a need for more effective AIT approaches. One of the most attractive is the use of synthetic peptides representing the B- or T-cell epitopes of the main allergens. This review summarizes experimental evidence of several T-cell epitopes derived from the Ole e 1 sequence to modulate the response to olive pollen in vitro, associated with several possible mechanisms that these peptides could be inducing, showing their usefulness as a safe preventive tool for these complex diseases.

## 1. Allergy and Tolerance

Allergic diseases, which are adverse reactions of the immune system (IS) to theoretically innocuous environmental antigens, are a global health problem that affects up to 30% of the population in industrialized societies [1,2], being an important cause of school and work absenteeism, with a great cost to health-systems and, therefore, a highly relevant public health problem.

The allergic response is mediated by the interaction between the antigen (allergen) and immunoglobulin E (IgE) antibodies bound to mast cells and basophils, which causes the release of inflammatory mediators that trigger clinical symptoms such as allergic rhinoconjunctivitis, asthma, skin inflammation, food allergy, and life-threatening anaphylactic shock. Nowadays, allergic diseases are considered a reflex of an immune response that has shifted toward type 2 inflammation. Briefly, after allergen exposure, epithelial-derived cytokines such as thymic stromal lymphopoietin (TSLP), IL-33 and IL-25 (called alarmins) induce the recruitment and activation of antigen presenting cells (APCs), including dendritic cells (DC) with a pro-allergic phenotype and type 2 innate lymphoid cells (ILC2). These cells secrete IL-4 and IL-13, promoting the polarization of naïve T cells toward Th2 cells, which produce IL-4, IL-5, IL-9 and IL-13. These cytokines will induce the production of allergen-specific IgE by B cells, the development and selective recruitment of eosinophils and basophils, the development of mast cells, airway hyperresponsiveness, and the production of mucus [3,4,5].

Tolerance of allergens by peripheral T cells seems crucial to a normal immune response. The immune regulatory response involves a complex system, which includes multiple populations of immunosuppressive cells such as myeloid-derived suppressor cells (MDSCs), regulatory B cells (Bregs), Natural Killer (NK) cells, immunosuppressive plasmocytes (ISPCs), and a regulatory subset of ILCs with interconnected functions that are currently being studied extensively [3,6,7,8]. Within this complex system, there are also subtypes of T cells with immunosuppressive function generically referred to as regulatory T cells (Tregs), wich have been described in humans, and play a fundamental role in allergen tolerance. Several types of Tregs cells have been defined based on their origin and categorized into two main groups: natural (nTregs) or induced (iTregs) [9]. nTregs or tymus-derived cells, express CD4, CD25, and Foxp3, and exhibit low expression of CD127 [10]. Natural regulatory T-cells often tend to tolerate self-antigens, exert their suppressive effects directly on cell–cell contact, and modulate allergen-specific T-cell responses in healthy nonatopic individuals [3,11,12]. In contrast, the iTregs cells or peripherally induced, are derived from peripheral lymphoid tissues [13] and mainly regulate immune responses against foreign antigens. iTregs include several distinct subsets: Foxp3-expressing iTregs, IL-10 secreting Tr1 cells, TGF-β producing Th3 cells [14,15,16], and IL-35-inducible regulatory T cells (iTR35), a newly identified subset of iTregs with potent immune regulatory properties [17], which are able to produce IL-10 and IL-35. Tregs act through at least four different suppressive mechanisms: secreting inhibitory cytokines including IL-10, TGF-β or IL-35; affecting cellular metabolisms by CD25, cAMP, adenosine receptor-2, histamine receptor (HR)-2, CD39, and CD73; suppressing antigen-presenting cells and ILCs by programmed cell death 1 (PD-1), cytotoxic T-lymphocyte associated protein 4 (CTLA-4), or inducible T cell costimulator ligand (ICOSL); and cytolysis mechanisms by granzymes A and B [6,18,19,20].

The role of the Tregs in modulating the type 2 inflammation induced in an allergic response has been one the main research focus for years. Pioneering works have shown how exposure to high levels of cat allergen was associated with a modified Th2 response [21]. Several studies in human peripheral blood mononuclear cells (PBMCs) have shown that allergen-specific Tregs play a role in maintaining immune tolerance, and these cells increase with the induction of natural tolerance in beekeepers during beekeeping season [22,23]. Also, our group demonstrated the involvement of regulatory mechanisms in the response to olive pollen, specifically a decrease in TGF-β and Foxp3 in patients allergic to olive pollen during the period of high pollen exposure and a recovery of the regulatory response, in allergic patients treated with specific immunotherapy to this pollen [24]. In fact, one of the most extensively studied parameters to determine tolerance induction during allergen-immunotherapy consists of monitoring the increase in Tregs during immunotherapy treatments [7].

## 2. Allergen-Specific Immunotherapy (AIT)

Currently, AIT is the only treatment capable of changing the course of the disease in patients with IgE-associated allergy [25]. AIT is considered a therapeutic vaccine that establishes tolerance against specific allergens [26]. It is based on the administration of the disease-causing allergens to induce a “counter immune response”. This response mainly consists in the production of allergen-specific IgG antibodies that can block IgE binding to allergens, and alterations in the cellular immune response, particularly a reduction of allergen-specific Th2 responses [27]. Vaccines currently used for AIT contain whole-protein allergens and are usually administered subcutaneously or sublingually, in increased doses until a maintenance dose is reached. Thus, tolerance induction is dependent on the immunogenicity and allergenicity of the allergen used as a vaccine. In fact, although the current AIT guidelines are effective, in some patients AIT may cause side effects that other formulations as modified proteins or peptides could avoid. In addition, the long-term use of these treatments (range from 3 to 5 years), can reduce treatment adherence. Therefore, there is a real need to improve AIT.

## 3. Peptide Allergen Immunotherapy: State of the Art

In the era of molecular diagnosis and treatments, new AIT approaches are being developed to decrease the allergenicity and adverse effects, as well as shorten the duration of the convectional AIT [28,29]. To achieve this, studies have been conducted on the use of hypoallergens, recombinant proteins, and methods other than subcutaneous and sublingual administration are under studying [30]. One of the most promising approaches is the use of peptides derived from the main allergens [20].

### Peptide Allergen Immunotherapy as a New Approach in AIT

This new therapy uses soluble synthesized allergen fragments of variable lengths and is based on the primary structure of the allergen. Depending on the length of the fragments and their ability to induce tolerance, peptide-based vaccines can be divided into those that use IgE-mediated peptides and other using T-cell peptides [31].

Vaccines designed using IgE-mediated peptides consist of long peptides (20–40 amino acids) that share main B-cell epitopes of the allergens; therefore, these peptides depend on the folded tertiary structure. They are usually synthesized with minor modifications or linked to a carrier protein to modify the humoral response in allergic patients. The ultimate goal is to induce a response by non-inflammatory allergen-specific antibodies (mainly IgG4) in order to block allergen-IgE binding [31,32]. On the other hand, the use of short synthetic peptides (10–17 amino acids), called T-cell peptides, is based on the lack of conformational B-cell epitopes. These short peptides are designed to be recognized by MHC class-II molecules on DC [33]. In theory, after peptide processing, the immune response should be led by Th1 and Treg responses, with a secretion of IL-10 that decreases eosinophils, basophils and mast-cells recruitment to the affected tissues and weakens the ability to release mediators. Moreover, this type of peptides is unable to bind to IgE- FcεRI on effector cells, mainly due to the small peptide size [20,34]. This characteristic confers an advantage over whole-allergen vaccines, due to the potential reduction of local and systemic adverse events, thereby leading to a better clinical outcome [33]. Peptide vaccines also offer additional advantages, such as high stability, easy purification, standardization and low production cost [35].

AIT with peptides has been studied for more than 20 years, and the first clinical trials were developed against cat allergy [36,37,38,39,40,41,42,43,44,45], house-dust-mites allergy (HDM) [46,47,48,49], and pollen allergies [50,51,52,53], but different effective clinical results have been obtained [54]. Regarding HDM, different types of peptides from Der p 1, one of the major allergens of *Dermatophagoides pteronyssinus*, have been analysed [46,47,48]. The most promising peptides combine a mixture of major Derp 1 epitopes, called HDM-SPIRE and have been tested in a phase II Clinical Trial. There was not reported any significant safety concerns. Patients treated with this drug reduced significantly the total rhinoconjunctivitis symptom score, compared to the placebo group. The most expective results were that the reduction of symptoms persisted 1 year after the treatment [49]. One of the most recent study that failed to achieve a clinically significant benefit for the treatment against cat allergies was a large-scale phase III study using a mix of peptides from Fel d 1 (major cat allergen) containing T-cell epitopes (Cat-PAD) capable of binding to commonly expressed class II HLA molecules [55]. This study was also evaluated in cat-allergic subjects who lived with a cat. The trial failed to demonstrate a treatment effect and found that peptide-based AIT was associated with an unusually high placebo response rate (approximately 60%), which may indicate a suboptimal study design [56]. However, a recent study based on a subgroup of patients from this trial described downregulation of the chemoattractant receptor-homologous molecule expressed on Th2 cells (CRTh2) in patients who had received the Fel d 1 peptide vaccine (Cat-PAD) [55], reporting no substantial deletion of allergen-specific CD4^+^ T cells. The authors concluded that the mechanisms of IT with peptide allergens could be different from the whole-allergen IT and suggested that the decrease of CRTh2 could result in a failure to recruit and activate these cells, thereby reducing Th2 inflammatory responses in the airways [56].

## 4. Olive Pollen Allergy

Olive (*Olea europaea*) pollen is one of the most important causes of respiratory allergy in the Mediterranean area and some regions of North America, South Africa, Japan, and Australia [57]. Specifically in Spain, it is the second most common cause of respiratory allergy after pollinosis to grass (approximately a 60% of all pollen-allergic patients are sensitized to olive pollen). Moreover, in certain areas of Andalusia (south of Spain), it is the most common respiratory allergy, with 84% of pollen-allergic subjects sensitized to olive pollen. In addition, the number of olive trees around the world is expected to increase considerably due to the health benefits of the Mediterranean diet, of which olive oil is a staple, thereby raising concerns that the number of patients with this pollinosis will increase worldwide [58].

At least 20 proteins with allergenic activity have been found in the olive pollen, and Ole e 1 is considered the major allergen. Almost of the 80% of olive-pollen allergic subjects show IgE specific to Ole e 1. It is a 145-aa glycoprotein with sequence microheterogeneity, highly dependent on the olive cultivar analyzed [59,60]. The importance of this allergen also resides in the high degree of sequence homology with other main allergens from the *Oleaceae* family as Fra e 1 (*Fraxinum*) and Syr v 1 (*Syringa*), which are responsible of a substantial percentage of pollinosis in Central Europe [61,62]. This high homology explains the cross-reactivity experienced by *Oleaceae*-allergic patients (IgE cross-reactivity greater than 80%). Ole e 1 is considered the main allergen of the *Oleaceae* family and has been described as a diagnostic marker of sensitization to pollens of this family, as well as other Ole e 1-like allergens such as Che a 1 from *Chenopodium album*, Lol p 11 from *Lolium perenne*, Pla l 1 from *Plantago lanceolate* and Phl p 11 from *Phleum pratense* [63]. Besides Ole e 1, a total of fifteen olive allergens (Ole e 1 to 15) have been characterized [62,64,65], several of which (e.g., Ole e 10, Ole e 7) are minority proteins in the whole-pollen extract though are major allergens in regions with extremely high antigenic load and are associated with the most severe symptoms of the disease (severe asthma) [66,67].

Currently, the standard treatment for olive-pollen-allergic patients is based on specific immunotherapy with whole-olive-pollen extracts. Despite improvements in the extracts used in AIT, the effectiveness of this strategy is highly variable, as it depends on the patient’s own sensitization, the severity of the clinical manifestations, and the treatment itself (the difficulty of standardizing allergenic extracts causes great variability in their therapeutic potential).

Besides, with AIT there is a risk that allergic patients may become sensitized to other components present in the extracts, an aspect of special relevance in olive pollinosis since minor components of the pollen (e.g., Ole e 10, Ole e 7) can be especially allergenic and could induce severe clinical symptoms in high doses. Consequently, the search for improvements to these types of vaccines is one of the most pressing objectives of research in the field of allergy. The design of new vaccines with allergen-derived peptides could offer multiple advantages as discussed above.

## 5. Ole e 1-Peptides

**Figure 1:**
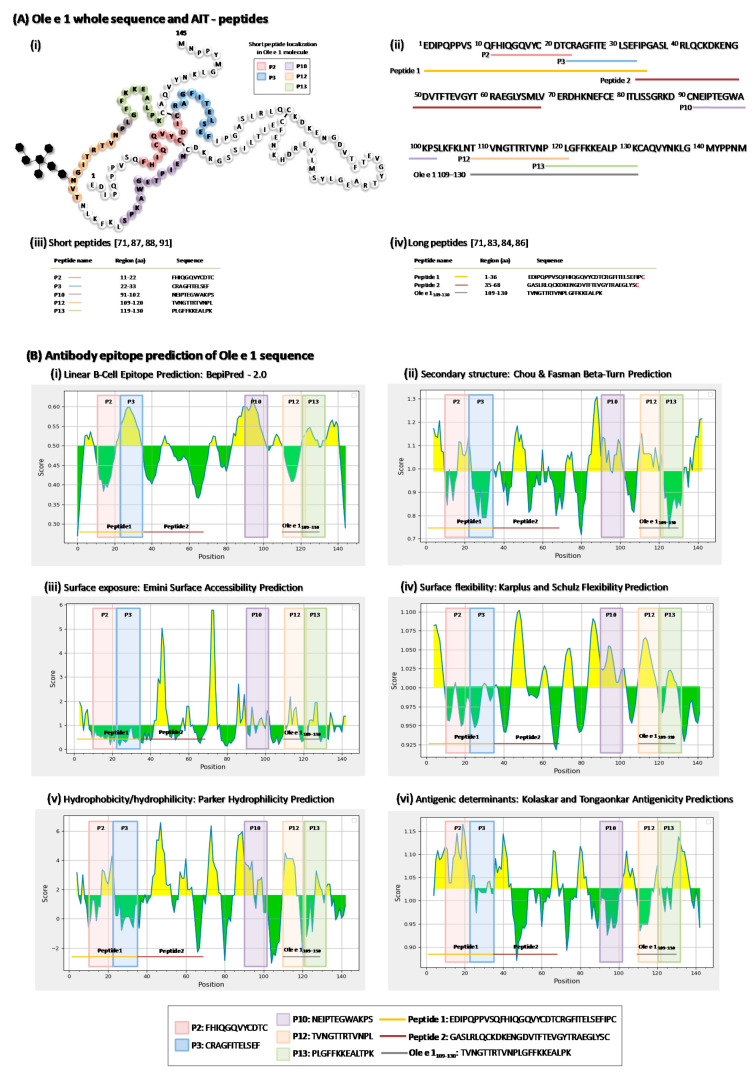
(**A**) Ole e 1 whole sequence and AIT peptides. (**i**,**ii**) Ole e 1 schematic and lineal sequence, respectively. In colors are highlighted the different peptides commented in this review. First, short peptides, or T-cell epitopes, most discussed in the review are highlighted in the schematic sequence in red (P2), blue (P3), purple (P10), orange (P12) and green (P13). On the linear sequence, both short and long peptides (B-cell epitopes) are highlighted, with a lot of sequence homology between both groups. (**iii**,**iv**) Short and long peptides relevant information, respectively: name, color legend, amino acid region within Ole e 1 whole-protein and peptide sequence. (**B**) Antibody epitope prediction of Ole e 1 sequence. Antibody or B-cell epitope in silico predictions evaluated by the IEDB server using the Ole e 1 sequence shown as imput sequence. (**i**–**vi**) Linear B-Cell epitopes, Chou and Fasman Beta-Turn, surface accessibility surface flexibility, Parker’s hydrophilicity and antigenicity predictions, respectively. This tool gives a probability score, represented in the *y*-axis, for each amino acid within the protein sequence, which positions are represented along the *x*-axis. The location of the peptides in the sequence is shown in the graphs, in order to identify the characteristics of the protein in the most immunomodulatory regions experimentally identified.

Ole e 1 is a 145-aa glycoprotein that was firstly sequenced by Edman degration [68], showing high microheterogeneity in several positions and 1 N-glycosylation site located at Asn-111 of the polypeptide chain (Figure 1A), which has been related with allergenic properties [69]. It has at least 4 B-cell epitopes [70] and 2 regions, aa 91 to 102 and aa 109 to 130, which were defined as immunodominant T-cell epitopes (later included in the Immune Epitope Database and Analysis Resource (IEDB), http://www.immuneepitope.org/refId/1005198, accessed on 20 May 2021), or regions mainly recognized by olive-pollen-allergic patients, able to induce in vitro a T cell-proliferative response with no IgE-binding capability [71].

### 5.1. Characterization of Ole e 1-Derived Peptides Immunoregulation

Up to now, different Ole e 1-derived peptides have been evaluated as AIT-peptides (Figure 1A). This review summarizes in greater depth the potential of short synthetic peptides, defined as T-cell epitopes, to prevent the response against the olive pollen in vitro, associated with evidence of possible mechanisms that these peptides could be modulating. However, other experimental approaches using long Ole e 1-derived peptides have been studied with very relevant results. Figure 1A summarizes the aa characteristics and location on Ole e 1 sequence from all Ole e 1-peptides used in this kind of studies (i–iv).

Also, thanks to the new bioinformatics tools based on multiparametric algorithms, it is possible to predict B- and T-cell epitopes based only on the protein sequence.

Figure 1B (i–iv) shows the B-cell epitopes prediction analysis (IEBD resource, accessed date 20 May 2021) for the Ole e 1 sequence, with the classical propensity scale methods, including BepiPred Linear Epitope Prediction 2.0 [72], Chou and Fasman Beta-Turn Prediction [73], Emini Surface Accessibility Prediction [74], Karplus and Schulz Flexibility Prediction [75], Parker Hydrophilicity Prediction [76], and Kolaskar and Tongaonkar Antigenicity [77]. All scores are based on properties of the amino acid sequence of the protein, providing information on the whole sequence in terms of possible B-cell epitopes, protein structure, exposure of each amino acid in the folded protein, flexibility, hydrophilicity (hence, exposure in biological fluids) and also, a simple antigenicity scale derived from physicochemical properties and amino acid frequencies in experimentally determined B cell epitopes [78]. This is just an in silico approach that provides information on the physical and chemical properties of a given sequence, and allows us to predict the regions that are theoretically best suited to be exposed and recognized by B cells, so they would be good candidates for immunomodulation. In this figure, we have also located within each graph the different Ole e 1 peptides (both long and short) used in AIT studies, to see if the sequence selection matches the prediction. In general, long peptides match regions predicted to be more suitable for possible B cell recognition. Short peptides are indicated in the figure only to locate them in sequence.

As well as with B-cell epitopes, a T-cell epitopes prediction analysis of Ole e 1 sequence was made, using the IEBD platform (accessed date 20 May 2021). T-cell epitope prediction aims to identify the shortest peptides within an antigen that are able to stimulate either CD4 or CD8 T-cells [79]. T-cell prediction methods aim to identify peptides within antigens that are immunogenic. T-cell epitope immunogenicity is based on three essential points: antigen processing, peptide binding to MHC molecules, and recognition by TCR. MHC-peptide binding is the most critical to determine T-cell epitopes [80,81]. Therefore, prediction of peptide-MHC binding is the main criterion used to anticipate T-cell epitopes. Here we have used the tool “T Cell Epitopes—Immunogenicity Prediction (IEBD)”, which predicts the relative ability of a peptide/MHC complex to elicit an immune response, and more specifically the analyses of “CD4 T cell immunogenicity prediction”, which combine CD4* T cell immunogenicity and HLA class II context [82]. Table 1 summarizes the best T-cell epitopes predicted by this bioinformatic model showing how the Ole e 1 peptides previously defined by our group (short peptides showed in Figure 1) [71] were predicted as T-cell epitopes, revealing that P3 and P12 had the best scores.

#### 5.1.1. Long Ole e 1-Derived Peptides

A long peptide including the immunodominant T-cell peptides (Ole e 1_109–130_) [71] was tested in a mouse model where a potential protection against olive-pollen sensitization was demonstrated, mainly by an increase of INF-γ (Th1 cytokine) and IL-10 (Treg cytokine) secretion [83]. Additionally, the same group used intranasal immunization with peptide-PGLA (polylactide-co-glycolide) microparticles, obtaining effective prevention of subsequent allergic sensitization to Ole e 1 in another murine model [84]. More recently, the use of dendrimeric scaffolds conjugated to this peptide has been investigated for the development of novel vaccines against olive pollen allergy. This conjugated promoted Tregs and IL10^+^ Tregs proliferation and IL-10 secretion by PBMCs from allergic patients [85].

In 2011, the IgE production capacity and the T-cell reactivity of 5 long Ole e 1-derived peptides (32 to 36 amino acids), compared to the whole sequence of Ole e 1, was studied by the group led by Valenta [86]. Rabbits were immunized with non-IgE-reactive, keyhole limpet hemocyanin- coupled peptides of Ole e 1. The results showed that there were 2 peptides from the N-terminus of the protein (Peptide 1 and Peptide 2 from Figure 1), which induced much lower lymphoproliferative responses than whole Ole e 1 [86].

#### 5.1.2. Short Ole e 1-Derived Peptides

Moreover, since the T-cell epitopes of Ole e 1 were defined [71], different basic and translational analyses have been performed. The basis of this review comes from the experimental definition of the T cell epitopes existing in the Ole e 1 molecule. This first work analyzed in vitro the proliferative response of PBMCs of healthy subjects and allergic to olive pollen patients against a battery of overlapping synthetic dodecapeptides (size appropriate for HLA recognition) covering the entire Ole e 1 sequence. Fifteen Ole e 1-derived dodecapeptides were studied. Peptides derived from the carboxyl terminal part of the Ole e 1 molecule, P10 (aa91–102), P12 (aa109–120) and, P13 (aa119–130) were described as T-cell immunodominant peptides, because they were mostly recognized by allergic subjects. On the other hand, in the amino terminal protein extreme, two peptides that included aa11–33Ole e 1 were mainly recognized by nonallergic subjects (P2 and P3; aa11–22 and 22–33, respectively) [71] and able to induce IL-10 cytokine secretion [87], being defined as potential immunoregulatory peptides.

Additionally, a more in-depth study was performed on the effect of these peptides on PBMCs from healthy controls and allergic patients collected inside and outside pollen season. There were temporal differences depending on the environmental allergen load. Thus, in PBMCs from patients collected during pollen season, stimulation with P2 and P3 increased IL-10 secretion significantly, compared with the same stimulus outside pollen season. In contrast, the control group showed a similar IL-10 secretion pattern irrespective of the pollen season. These results demonstrated the importance of taking into account the load pollen exposure in seasonal allergies to design an immunotherapy strategy. Results of Th1 and Th2 cytokine secretion were inconclusive. There was not a clear differential pattern between peptide treatments comparing healthy control vs. allergic groups [88].

### 5.2. Ole e 1 T Cell Peptides: Mechanisms of Action

#### 5.2.1. The Use of Peptides in the Era of Precision Medicine

The new era of precision medicine could provide specific details of molecular mechanisms involved in the immune system (IS), mainly though the development of –omics approaches [89,90]. In this sense, an extensive study was developed about how Ole e 1-peptides could act on the gene expression and biological processes in olive-allergic patients compared to nonallergic subjects. The main molecular pathways involved in the Ole e 1 peptides (P2, P3 and P10, P12 and P13) response referred to the “antigen presentation and processing” pathway, however not “B cell receptor signaling” pathway was altered as was found with the whole allergen extract of *Olea europaea* pollen. Pathways related to “endothelial migration” and “molecular adhesion” with functional pathways as asthma were over-activated with the *Olea* extract and P10, P12 P13 but not with the immunomodulatory P2 and P3. [88]. All these data reinforce the idea that the use of bioinformatics approaches could aid in the clinical management of allergic diseases. Apart from pathway and biological-process analysis, our group was able to define and validate a set of 51 genes that were modulated by P2 and P3. The major part of these genes are involves in the immune system regulation: EBI3, the subunit of IL-35 related to regulation of the immune response; DAB2, a FOXP3 target gene required for Treg; LGMN, a gene implicated in human Treg cell function, which is induced upon T-cell receptor stimulation; DMT1, enzyme associated with DNA methylation; CD84, a leukocyte receptor that may modulate FcεR-mediated signaling and plays a protective role against allergic response and MIR 155 (epigenetic regulator). Also, two genes which has been described as candidate biomarkers of early efficacy of allergen immunotherapy, C1Q and STAB1 [88] were also found. Finally, were included in this set of genes modulated by P2 and P3 other attractive genes such as FPR3, an innate immune receptor that could be implicated in nasal tissue remodeling; TREM1, ALOX5, PTGS2 and several cytokines, chemokines, and adhesion molecule ligands (CCL20, CCL22 etc.). These findings could lead to new treatments, though not only for olive allergy, since there are great similarities between olive pollen and other *Oleaceae*-family pollens, as discussed before. Thus, this therapeutic tool could be useful in the treatment of several allergies [88].

#### 5.2.2. Cellular Response: Treg vs. Th2 Response

In our most recent publication, we evaluated the in vitro ability of Peptides 2 and 3 (P2+P3) and Peptides 10, 12 and 13 (P10+P12+P13) in combination to modulate the allergen-specific response to whole-olive-pollen extract. We hypothesized that the combination of all of them would be a good therapeutic alternative for this disease. P10, P12 and P13 were thought to act as immunogens (specific to the allergic response), while P2 and P3 may modulate the immune response toward a tolerogenic response, as long as none of the combinations were allergenic. To achieve this, we performed in vitro analyses to determine the ability of combinations of Ole e 1 immunomodulatory peptides (immunoregulatory and immunodominant peptides) to prevent or reverse the olive-pollen response of PBMCs from allergic and nonallergic subjects. Also, the safety of these peptides was determined as the absence of basophil activation [91]. In this study, besides the PBMCs proliferation and the study of classical regulatory cytokine, IL-10, a new regulatory cytokine, IL-35, was also analyzed.

The main results of this work confirmed that P2 and P3 are able to partially prevent the proliferative response against whole-olive-pollen extract but are not capable of reversing a previously established response. Their combination with immunodominant peptides increased the preventive response, from 22.8% when assayed alone to 32.7% when used in combination. Also, PBMCs from patients with asthma showed the best prevention with these peptides, showing the highest decrease in the response against to olive-pollen-extract after being pre-stimulated with peptides.

**Figure 2:**
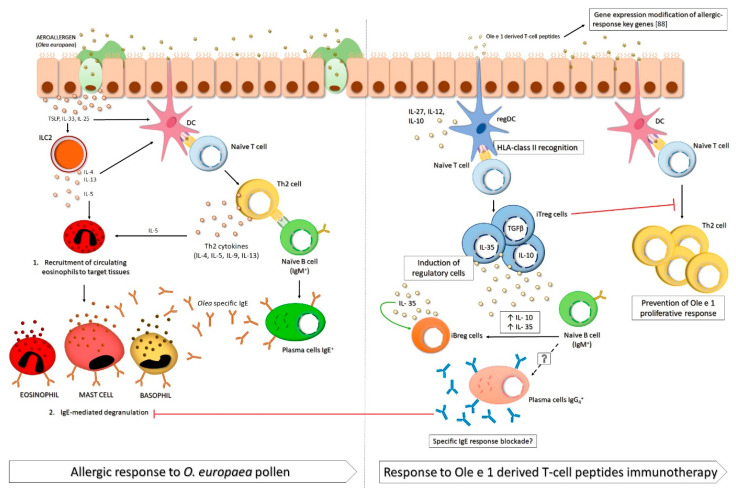
Schematic representation of the allergic response to *Olea europaea* pollen and theoretical effects of Ole e 1-derived peptide immunotherapy. (**Left side**). Simplified scheme of the immune response to aeroallergens (i.e., *Olea europaea* pollen). First, the respiratory epithelium of allergic patients, in response to an allergen, secretes molecules such as TSLP, IL-33, or IL-25, which activate type 2 innate lymphoid cells (ILC2). Then, these cells release interleukins such as IL-4, IL-5, and IL-13, which, along with epithelial-secreted molecules, activate dendritic cells (DCs). Activated DCs internalize the allergens and present them to naïve T-cells, which proliferate to Th2 cells. This Th2 response is characterized by a Th2-cytokine secretion (IL-4, IL-5, IL-9, and IL-13) that induces the recruitment of circulating eosinophils to target tissues. Also, Th2-lymphocytes present allergens to IgM+-naïve B-cells, which differentiate into IgE+ plasma cells. These cells secrete allergen-specific IgE, which binds to the mast cells, basophils, and eosinophils in tissue, ultimately releasing inflammatory mediators. (**Right side**). Simplified scheme of the theoretical regulatory immune response to immunotherapy with Ole e 1 T-cell peptides, in accordance with our previous results. The exposure of allergic respiratory epithelium to T-cell peptides leads to a regulatory DC (regDC) activation that, through HLA-class II recognition, induce the expansion of regulatory cells. First, regDC presents peptides to naïve T-cells, which differentiates into induced regulatory T-cells (iTreg). Within these cells, IL-10, TGFβ, and IL-35-secretory iTreg cells are most important. These regulatory cytokines, specifically IL-10 and IL-35, promote the maturation of naïve B-cells to iBreg cells, which secrete more IL-35, in autocrine immune signaling. Also, we hypothesize that naïve B-cells also differentiate into IgG4^+^ plasma cells, which secrete blocking IgG4 antibodies with the ability to block specific IgE response. Furthermore, in a future contact with the whole allergen, the iTreg cells produced by immunotherapy, may prevent the proliferative response of Th2 cells. Last, we observed a gene-expression modification of key allergic-response genes in PBMCs from allergic patients in response to stimulation with T-cell peptides.

According to cytokine results, P2 and P3-alone or in combination with immunodominant peptides-induced IL-10 and very high IL-35 secretion levels, improving the microenvironment which benefits immunotolerance in untreated allergic subjects. Additionally, in concordance with the proliferation assay results, these peptides added after olive-pollen extract, were unable to induce the secretion of regulatory cytokines. Overall, these data highlight the fact that Ole e 1 peptides could be usefulness as preventive tools for this allergy. They induce a regulatory response mediated by IL-35 and IL-10, which is able to reduce the later response against to olive pollen. These data are in agreement with the idea that several authors [92,93] propose, i.e., the prophylactic usefulness of T-cell vaccines. Figure 2 summarizes the potential of Ole e 1-derived T cell epitopes for their use as AIT.

The results of IL-35 were especially interesting for being a novel regulatory cytokine recently proposed as a potential new biomarker of AIT [94], with immunoregulatory properties. IL-35 is a member of the IL-12 family, which is mainly secreted by stimulated Tregs [95]. It is an heterodimer which consists of an EBI3 subunit and an IL-12A (also known as p35) subunit [96]. In contrast to IL-12, IL-23, IL-27 (the rest of the IL-12 family) which are involved in the pro-inflammatory response, IL-35 suppress the inflammatory immune response. In this study, IL-35 was included because EBI3 was one of the genes previously found to be specifically modulated by P2 and P3 and considered as a possible therapeutic target for olive-pollen allergy [88].

The immunosuppressive activity in different inflammatory and autoimmune diseases of IL-35 is being extensively studied, mainly in animal models [97,98,99]. IL-35 promotes the development of Tregs and Bregs and, recently, has been described the correlation between IL-10 and IL-35, by the STAT1 and STAT3 phosphorylation in B cells, promoted by IL-35 [100]. Although there are few studies of IL-35 in relation with allergic diseases, their interest is increasing in the last years [94]. The abnormal expression of IL-35 in asthma was described, suggesting an important role of this cytokine in the pathogenesis asthma [101]. It has also been described the low levels of this cytokine in people with asthma and chronic obstructive pulmonary disease (COPD), though an increase in serum levels of the cytokine is observed after immunotherapy, associated with an improvement in clinical symptoms [102].

In accordance with our results, very recently [94] IL-35 and iTreg 35 cells have been described as being induced by sublingual allergy immunotherapy, suggesting that IL-35 therapy could be useful for the treatment of respiratory allergic diseases. Indeed, IL-35 has been found to be able to inhibit IgE production by B cells [94]. This aspect could be especially relevant considering that one of the main weaknesses of short-peptides treatments is the inability to reduce the IgE antibody response by IgG4 antibody induction. The fact that Ole e 1 regulatory peptides induce IL10, but mainly high IL-35 secretion, is very promising, and needs to be studied in depth. Besides, due to this high IL-35 induction in response to P2+3, but not to P10+12+13 or even the whole olive pollen extract, it would be interesting to deepen in the effect of these peptides in the STAT1/STAT4 signaling, since it has recently been shown that STAT1:STAT4 heterodimer is an essential effector of IL-35 [103].

Finally, to broaden the knowledge about the behavior of the immunoregulatory peptides, an analysis of P2 and P3 in “The Eukaryotic Linear Motif resource for Functional Sites in Proteins (ELM)” a platform specialized in database of peptides/proteins and functionality (http://elm.eu.org/, accessed on 20 May 2021), was performed. In this analysis, also the immunodominant peptides, P10, P12 and P13; were included. Results are summarized in Table 2. In respect to P2 and P3, both peptides showed functional motifs related with the protein stability, a N-degron motif or destabilizing N-terminal residue that permit the ubiquitin-dependent proteasomal degradation, Peptide 2 by N-box domains and Peptide 3 by UBR-box. Also, both showed predicted PDZ domain ligands, short C-terminal peptides that bind in a surface groove of PDZ domains of proteins as a part of a variety of biological processes including cell signaling and synapse. There are different classes according pattern of recognition (Table 2). P2 showed a PDZ domain ligand of class 3 and P3 of class 1. Finally, the most remarkable result was the finding of a matched sequence in P2 with a phosphotyrosine ligands bound by SH2 domains defined as the STAT5 Src Homology 2 (SH2) domain binding motif. STAT5 has been shown to be essential for nTreg development [104,105,106] and several reports have found an association between the increase of IL-35 and STAT5 [107]. Correlation among Ole e 1-Peptide 2/STAT5 and IL-35 could have relevant functional implications that may be useful in allergic treatments. In the case of P10, P12 and P13, there are mainly functional motifs related to post-transcriptional modifications.

## 6. Conclusions

In conclusion, experimental models and clinical trials have demonstrated the potential usefulness of peptides derived from main allergens as a promising tool for the treatment of wide type of allergies. Although the underlying immunological mechanisms are not currently well understood, peptide immunotherapy appears to be dependent upon IL-10 and other Tregs mediators. In the specific case of olive pollen, the use of peptides has demonstrated to regulate genes and routes of activation that are involved in the tolerogenic response. In this sense, the combination of five short dodecapeptides Ole e 1 derived-peptides is able to prevent the olive-pollen proliferative response associated to IL-10 and IL-35 regulatory cytokines production in allergic patients. Moreover, these combinations of peptides are not capable of inducing basophils activation, a pre-requisite for the development of a new peptide vaccine. Further basic and clinical studies are needed to broad knowledge of these capacities and to confirm their possible use in routine clinical practice.

## Figures and Tables

**Table 1:** CD4 Immunogenicity prediction results.

	Peptide	Start	End	Combined Score	Immunogenicity Score	Peptide Core	Median Percentile Rank (7-Allele)	HLA-DRB1: 03:01	HLA-DRB1: 07:01	HLA-DRB1: 15:01	HLA-DRB3: 01:01	HLA-DRB3: 02:02	HLA-DRB4: 01:01	HLA-DRB5: 01:01
Ole e 1 sequence	TCRAGFITELSEFIP	21	35	56.25	97.12	FITELSEFI	29.0	55.0	20.0	24.0	3.1	31.0	51.0	29.0
	SEFIPGASVRLQCRE	31	45	56.20	98.51	FIPGASVRL	28.0	46.0	9.1	28.0	34.0	3.9	42.0	19.0
	VGYTRAEGLYSMLVE	56	70	58.36	99.42	YTRAEGLYS	31.0	71.0	17.0	33.0	31.0	15.0	64.0	11.0
	EFCEITLISSGRKDC	76	90	57.37	90.93	ITLISSGRK	35.0	35.0	35.0	11.0	83.0	39.0	58.0	0.9
	PSLKFILNTVNGTTR	101	115	35.37	71.92	LKFILNTVN	11.0	27.0	18.0	14.0	8.4	0.01	11.0	7.0

Results obtained with the whole Ole e 1 sequence in the CD4 immunogenicity prediction tool [82], using the combined method with a 60% threshold. In the peptide sequence, the overlapping with Ole e 1 T-cell peptides previously described by our group [71] are highlighted in red for P2, blue for P3, purple for P10 and orange for P12.

**Table 2:** Eukaryotic Linear Motif (ELM) in sequence of P2, P3, P10, P12 and P13.

Elm Name	Name	Matched Sequence	Positions	ELM Description	Cell Compartment	Pattern	Probability
DEG_Nend_Nbox_1	N-degron	FHIQGQVYCDTC	1-2	N-terminal motif that initiates protein degradation.	Cytosol	^M{0,1}[FYLIW][^P]	2.302 × 10^−4^
LIG_PDZ_Class_3	Class 3 PDZ domain ligands	FHIQGQVYCDTC	7–12	C-terminal class 3 PDZ-binding motif.	Cytosol, internal side of plasma membrane	...[DE].[ACVILF]$	6.168 × 10^−5^
LIG_SH2_STAT5	STAT5 Src Homology 2 (SH2) domain ligand	FHIQGQVYCDTC	8–11	STAT5 Src Homology 2 (SH2) domain binding motif.	Cytosol	(Y)[VLTFIC]..	3.296 × 10^−3^
DEG_Nend_UBRbox_4	N-degron	CRAGFITELSEF	1–2	N-terminal motif that initiates protein degradation.	Cytosol	^M{0,1}(C).	1.768 × 10^−5^
LIG_PDZ_Class_1	Class 1 PDZ domain ligands	CRAGFITELSEF	7–12	C-terminal class 1 PDZ-binding motif.	Cytosol, internal side of plasma membrane	...[ST].[ACVILF]$	7.255 × 10^−5^
DEG_Nend_UBRbox_3	N-degron	NEIPTEGWAKPS	1–2	N-terminal motif that initiates protein degradation.	Cytosol	^M{0,1}([NQ]).	1.645 × 10^−4^
LIG_LIR_Apic_2	LC3-interacting region ligand	NEIPTEGWAKPS NEIPTEGWAKPS	5–11 6–11	Apicomplexa specific variant of the canonical LIR motif involved in autophagy.	Cytosol, cytoplasmic side of late endosome membrane	[EDST].{0,2}[WFY]..P	3.371 × 10^−3^
LIG_FHA_1	FHA phosphopeptide ligands	TVNGTTRTVNPL	4–10	Phosphothreonine motif binding a subset of FHA domains.	Nucleus	..(T)..[ILV].	8.662 × 10^−3^
MOD_N-GLC_1	Generic motif for N-glycosylation	TVNGTTRTVNPL	2–7	Generic motif for N-glycosylation.	Extracellular, Golgi apparatus, endoplasmic reticulum	.(N)[^P][ST]..	5.018 × 10^−3^
CLV_PCSK_SKI1_1	Subtilisin/kexin isozyme-1 (SKI1) cleavage site	PLGFFKKEALPK	7–11	Subtilisin/kexin isozyme-1 (SKI1) cleavage site.	Endoplasmic reticulum lumen, endoplasmic reticulum, Golgi apparatus, extracellular	[RK].[AILMFV][LTKF].	6.821 × 10^−3^
LIG_REV1ctd_RIR_1	Rev1-interacting regions ligand	PLGFFKKEALPK	2–10	DNA repair proteins interact with the C-terminal domain of the Rev1 translesion synthesis scaffold (Rev1-Interacting Region).	Nucleoplasm, nucleus	..FF[^P]{0,2}[KR]{1,2}[^P]{0,4}	5.350 × 10^−4^
MOD_SUMO_for_1	Motif recognised for modification by SUMO-1	PLGFFKKEALPK	5–8	Motif recognised for modification by SUMO-1	Nucleus, PML body	[VILMAFP](K).E	1.914 × 10^−3^

Sequence of P2, P3, P10, P12 and P13 are analyzed with an ELM prediction tool (http://elm.eu.org/, accessed on 20 May 2021) which shows concordant motifs between the introduced sequence and common eukaryotic linear motifs. The results for sequence of P2, P3, P10, P12 and P13 are shown in red, blue, purple, orange and green, respectively.

## Data Availability

Not applicable.
